# Identifying and quantifying initial post-discharge needs for clinical review of sick, newborns in Kenya based on a large multi-site, retrospective cohort study

**DOI:** 10.3389/fped.2024.1374629

**Published:** 2024-09-26

**Authors:** John Wainaina, Esther Lee, Grace Irimu, Jalemba Aluvaala, Mike English, The Clinical Information Network Author Group, Dolphine Mochache

**Affiliations:** ^1^ Health Services Unit, KEMRI-Wellcome Trust Research Programme, Nairobi, Kenya; ^2^ Department of Pediatrics, Rush University Medical Center, Chicago, IL, United States; ^3^ Department of Paediatrics and Child Health, University of Nairobi, Nairobi, Kenya; ^4^ Nuffield Department of Clinical Medicine, Oxford, United Kingdom

**Keywords:** newborn, infant, post-discharge, specialist, follow-up, morbidity, complications

## Abstract

**Background:**

Progress in neonatal care has resulted in a 51% decrease in global neonatal mortality rates from 1990 to 2017. Enhanced survival will put pressure on health care systems to provide appropriate post-discharge, follow-up care but the scale of need for such care is poorly defined.

**Methods:**

We conducted a retrospective cohort study of newborns discharged from 23 public hospital neonatal units (NBUs) in Kenya between January 2018 and June 2023 to identify initial follow-up needs. We first determined pragmatic follow-up categories based on survivors’ clinical conditions and morbidities. We then used individual phenotypes of individual babies to assign them to needing one or more forms of specialized clinical follow-up. We use descriptive statistics to estimate proportions of those with specific needs and patterns of need.

**Findings:**

Among 136,249/159,792 (85.3%) neonates discharged, around one-third (33%) were low birth weight (<2,500 g), and a similar 33.4% were preterm (<37 weeks). We estimated 131,351 initial episodes of follow-up would be needed across nine distinct follow-up categories: general pediatrics, nutrition, growth & development (40.4%), auditory screening (38.8%), ophthalmology for retinopathy of prematurity (9.6%), neurology (8.0%), occupational therapy (1.3%), specialized nutrition (0.9%), surgery (0.8%), cardiology (0.2%), and pulmonary (<0.1%). Most neonates met the criteria for two (52.3%, 28,733), followed by three (39.6%, 21,738) and one follow-up episodes (5.6%, 3,098). In addition to prematurity and very low birth weight (≤1,500 g), severe infections with extended gentamicin treatment, severe jaundice managed with phototherapy, and hypoxic-ischemic encephalopathy (HIE) contributed substantially to the pattern of need for post-discharge follow-up.

**Conclusions:**

Almost half of surviving NBU infants have multiple specialty post-discharge follow-up needs. More urgent attention needs to be focused on healthcare planning now to guide strategies to address the varied medical and developmental needs that we outline in resource-constrained contexts like Kenya.

## Introduction

Advances in antenatal and neonatal intensive care, together with global efforts to improve outcomes for small and sick newborns, have transformed the care and survival of newborns. Between 1990 and 2017, there was a 51% decrease in the global neonatal mortality rate from 37 to 18 per 1,000 live births (1–4). In Kenya, neonatal mortality rates have exhibited a modest decline, with 20 neonatal deaths per 1,000 live births reported in 2022, a slight decrease from 22 deaths per 1,000 live births in 2014 (5, 6). As a result, newborns are now more likely to survive prematurity and early neonatal morbidities such as severe perinatal asphyxia, serious infections, or with congenital malformations (2–4).

Complications of prematurity and serious conditions in the first days of life include cerebral palsy, blindness, hearing impairment, learning disabilities, impaired growth, and chronic respiratory disease (7–10). The World Health Organization (WHO) estimates there were about 15 million preterm births in 2020 (11, 12), with 81% of these being in Low and Middle-Income Countries (LMICs). The need for follow-up care to ameliorate the longer-term burden of post-neonatal mortality and morbidity are therefore most pronounced in these countries (13, 14). Yet LMICs are poorly prepared to provide such care, while even in Middle-Income Country (MIC) settings, the risk of disability for infants born between 28 and 32 weeks of gestation may be double that of High-Income Countries (HICs) (15).

We therefore stand at a critical juncture. As we improve global neonatal survival, we must develop health services that provide quality follow-up care for surviving neonates to optimize their long-term outcomes. However, most LMICs, including Kenya, lack information on the burden of post-neonatal morbidities to inform such planning. We aimed to address this gap by examining NBU survivors’ discharge treatment and morbidity patterns to begin to determine the likely scale and scope of follow-up needs.

## Materials and methods

### Study design

We conducted a retrospective cohort study of discharged newborns between January 2018 to June 2023 from 23 public, Clinical Information Network-Neonatal (CIN-N) participating hospitals across 19 counties in Kenya. Twenty-two of the hospitals are county referral hospitals serving their local populations. They aim to provide intermediate newborn care, which includes Continuous Positive Airway Pressure (CPAP), oxygen therapy, intravenous fluids, antibiotics, and phototherapy amongst other interventions (16). We also included one tertiary NBU that provides mechanical ventilation, surfactant treatment, surgical, and other sub-specialty services (16). The CIN was formed in 2013 through collaboration between the Ministry of Health, KEMRI Wellcome Trust Research Programme, Kenya Paediatric Association/Kenya Paediatric Research Consortium, the University of Nairobi, and the participating hospitals (17, 18). Ethical approval for this work was provided by KEMRI's Scientific and Ethical Review Unit (KEMRI/RES/7/3/1 SSC PROTOCOL No. 2465).

Trained Health Records and Information Officers extracted patient information from inpatient medical records into a standardized Research Electronic Data Capture (REDCap) platform (19) at the time of a baby's discharge. Essential data, including demographic information, maternal and patient history, clinical characteristics, admission and discharge diagnoses, treatments, and supportive care are captured (19).

### Categorization of morbidities

With the collective input of all authors, including four experienced pediatric/neonatal practitioners (JA, EL, GI, and ME), we systematically classified key diagnoses observed throughout the admission process. This classification was structured in a problem-based manner, allowing us to effectively allocate surviving newborns to one or more pragmatically created follow-up care categories. In essence, this pragmatic framework for categorizing morbidities was informed by multiple sources, including (1) documented diagnoses and clinical characteristics, (2) clinical expertise, (3) contextual knowledge gained through discussions with hospital teams during Clinical Information Network annual meetings, and (4) collaborative discussion and consensus among the authors. We could not use a baby's actual follow-up plans as in Kenya most facilities lack access to specialist services other than post-discharge referral to the general pediatric or neonatal outpatient clinic. Such clinics typically do not provide multi-professional team support from trained nurses, physiotherapists, occupational therapists, or nutritionists.

**Figure 1:**
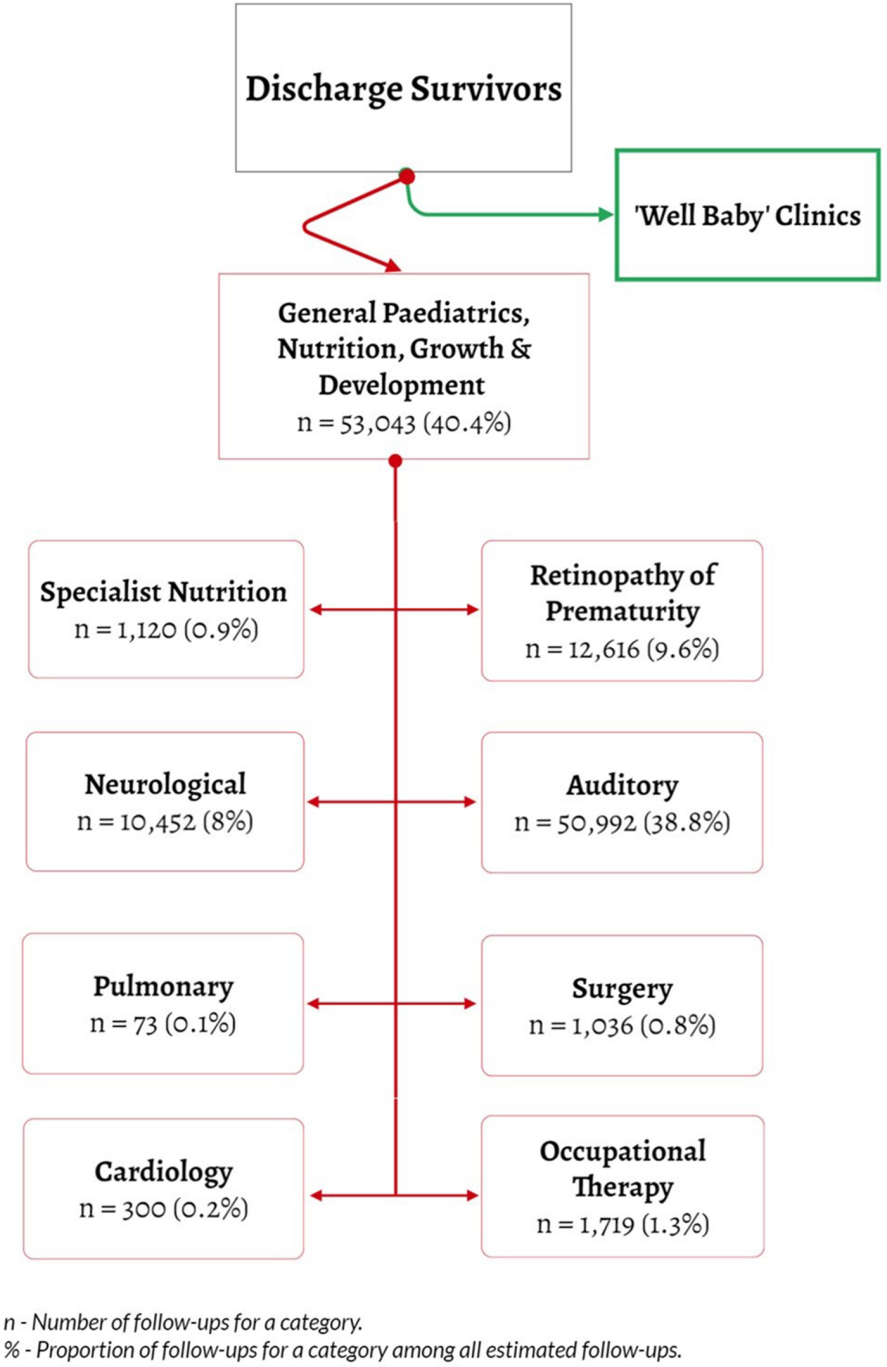
Flow of neonatal patients at discharge and subspeciality follow up categories for at risk post-discharge.

We created eight distinct follow-up categories. These aimed to balance expected need for specific knowledge and skills of providers and feasibility of meeting such skills needs in the medium-term. Follow-up categories were Neurology, Specialist Nutrition, Auditory Screening, Ophthalmology/Retinopathy of Prematurity (ROP), Pediatric Orthopedic and Occupational Therapy, Cardiology, Pulmonary, and (Paediatric) Surgical Review. A ninth category indicated the coordinating role of a pediatrician who could also assess the necessity for additional specialized follow-up care not immediately apparent at the point of discharge, or if the infants could benefit from paediatrician's assessment and triaging for sooner specialty appointment needs. The paediatrician would also provide ongoing general paediatric care together with nutritional support and monitoring of growth and development post-discharge for the high-risk population. Low-risk babies at discharge might only require “well-baby clinic” follow-up typically provided by nurses encompassing growth monitoring, simple checks on developmental milestones and immunization (Figure 1).

We used data on admission and discharge diagnoses, interventions received by NBU survivors, their individual characteristics, and documented morbidities (considered as discharge “phenotypes”) to logically allocate them to one or more of the nine follow-up categories (Table 1). Certain morbidities that necessitate surgical interventions, such as facial/lip cleft, spina bifida and hernia, were allocated first to general pediatric follow-up while awaiting paediatric surgical referral. This decision was grounded in the understanding that specialist paediatric operative surgical care is not typically available during the initial admission or for local follow-up in county hospitals. However, emergent cases that necessitated transfer would have been initiated to the highest-level facility. General paediatricians will likely first provide an initial comprehensive post-discharge assessment as these visits are often scheduled within two weeks of discharge and help evaluate and advocate for the timing and urgency of paediatric surgical referral.

**Table 1:** Subspeciality follow-up needs for babies with birth weight and or gestation risk, diagnosis, and or intervention morbidity risk indications.

Phenotype	Neurology	Specialist nutrition	Auditory screening	Ophthalmology- ROP	Pulmonary	Pediatric orthopedic and occupational therapy	Cardiology	Surgical	General pediatrics, nutrition, growth & development
Birth weight ≤1,500 g/<32 weeks			X	X					X
Mechanical ventilation				X	X				X
Bronchopulmonary dysplasia				X	X				X
Convulsions/seizures	X		X						X
SBA/HIE	X		X						X
Sepsis/serious bacterial infection			X						
Neonatal tetanus	X		X						
Neonatal meningitis	X		X						
Patent Ductus Arteriosus (PDA)							X		X
Pulmonary hypertension							X		X
Kidney injury									X
Hydronephrosis									X
Necrotizing Enterocolitis (NEC)		X							X
RH/ABO isoimmunization			X						
HIV/VDRL exposure									
Hypoglycemia									X
Severe jaundice									X
Phototherapy treated			X						
Exchange transfusion									X
Kernicterus	X		X	X					X
CTEV/club foot						X			
Birth/tissue injury						X			
Paralysis	X					X			
Down syndrome									X
Facial/Lip cleft	X	X	X					X	X
Congenital deformities^a^									X
Gastroschisis		X						X	X
Spina Bifida								X	X
Hernia									X
Hirschsprung disease		X							X

ROP, retinopathy of prematurity; SBA, severe birth asphyxia; HIE, hypoxic ischaemic encephalopathy.

^a^ Not specified | HIV – Human Immunodeficiency Virus | VDRL - Venereal Disease Research Laboratory | CTEV – Congenital Talipes Equinovarus.

Our overall aim in creating these categories was to use data to estimate the need for improved post-neonatal follow-up services in Kenyan county hospitals in the medium term. These might be more limited than those on offer in a highly resourced, technologically advanced health system such as the United States, where for example, virtually all newborns are screened for hearing loss before leaving the hospital (20). As such, our estimations of follow-up needs might be regarded as a minimum requirement to target initial resource use rather than immediately progressing to approaches based on universal screening.

### Data analysis

We utilize descriptive statistics to present a comprehensive overview of the demographic and clinical characteristics of the sample. Using pooled data, we calculated frequencies and proportions as estimates of initial follow-up needs and examined morbidities contributing to these follow-up requirements. Our focus was only on initial follow-up needs, we made no attempt to estimate subsequent follow-up visit frequency over the longer term. We used R Statistical Programming software version 4.3.3 for all analysis.

## Results

159,792 newborns were admitted to the Newborn Units (NBUs) of 23 CIN-N hospitals between January 2018 and June 2023. 136,249 individuals (85.3%), including 7,809 (5.2%) from the tertiary hospital survived until discharge and were included in the analysis. Sex was documented for 135,423 neonates (99.4%), with 75,717 (56%) identified as male. Within the sample, 134,474 (98.7%) birth weights (BW) and 117,524 (86.3%) gestational ages (GA) were recorded with 44,386 (33.0%) low birth weight (<2,500 g) and 39,289 (33.4%) preterm births (<37 weeks). In the total cohort, 15,714 newborns (12%) were referrals to the participating CIN-N units, showing variability among hospitals (ranging from 1.1% to 34.1% for county hospitals and 17% for the tertiary hospital). Among these admissions, 2,146 newborns (1.6% of total cases) were referred out to higher-level facilities, with inter-hospital rates among county hospitals ranging from 0.5% to 4.7% (and 0.0% for the tertiary hospital) Table 2. The proportions receiving specific interventions (e.g., CPAP and phototherapy) are also presented in Table 3.

**Table 2:** Demographic and clinical characteristics of discharged newborns (*N* = 136,249).

Characteristic	Documented, *N* [%]	Missing, *N* [%]	*N* (%)
Sex	135,423 [99.4]	826 [0.6]	
Female			59,659 (44%)
Male Indeterminate			75,717 (56%) 47 (<0.1%)
Birth weight (grams)	134,474[98.7]	1,775 [1.3]	
<1,000			823 (0.6%)
1,000–1,499			7,312 (5.4%)
1,500–1,999			17,896 (13%)
2,000–2,499			18,355 (14%)
2,500–4,000			82,661 (61%)
>4,000			7,427 (5.5%)
Gestation (weeks)	117,524 [86.3]	18,725 [13.7]	
Extreme preterm (<28)			1,754 (1.5%)
Very preterm (28 ^0/7^–31 ^6/7^)			8,793 (7.5%)
Moderate preterm (32 ^0/7–^33 ^6/7^)			8,862 (7.5%)
Late preterm (34 ^0/7^–36 ^6/7^)			19,880 (16.9%)
Term (≥37)			78,235 (66.6%)
Place of birth	136,249 [100.0]	0 [0.0]	
Inborn			120,535 (88%)
Referred in			15,714 (12%)
Day of admission	136,248 [100.0]	1 [<0.1]	
After day of birth			35,370 (26%)
Day of birth			100,878 (74%)
Prescribed gentamicin	110,638 [81.2]	25,611 [18.8]	
No			37,611 (34%)
Yes			73,027 (66%)
Administered gentamicin (days)	69,419 [51.0]	66,830 [49.0]	
≤3			33,697 (49%)
≥4			35,722 (51%)
Complicated Prematurity^a^	136,249 [100.0]	0 [<0.1]	
No			114,661 (84%)
Yes			21,588 (16%)
Administered Oxygen	69,331 [50.9]	66,918 [49.1]	
No			39,157 (56%)
Yes			30,174 (44%)
Given CPAP	117,590 [86.3]	18,659 [13.7]	
No			112,016 (95%)
Yes			5,574 (4.7%)
Treated with Phototherapy	136,249 [100.0]	0 [0.0]	
No			103,015 (76%)
Yes			33,115 (24%)
Destination at Discharge^b^	136,249 [100.0]	0 [0.0]	
Alive Home			134,103 (98%)
Referred Out			2,146 (1.6%)

CPAP, continuous positive airway pressure.

^a^ Complicated prematurity – prematurity and any other diagnosis.

^b^ Study population included NBU admissions who survived.

**Table 3:** Distribution of follow-ups among all survivors.

Follow up	Counts (*N*)	%	Counts per year
General pediatrics, nutrition, growth & development	53,043	40.4%	9,644
Auditory screening	50,992	38.8%	9,271
Retinopathy of prematurity	12,616	9.6%	2,294
Neurology	10,452	8.0%	1,900
Occupational therapy	1,719	1.3%	313
Specialist nutrition	1,120	0.9%	204
Paediatric surgical review	1,036	0.8%	188
Cardiology	300	0.2%	55
Pulmonary	73	0.1%	13

### Follow-up categories

**Figure 2:**
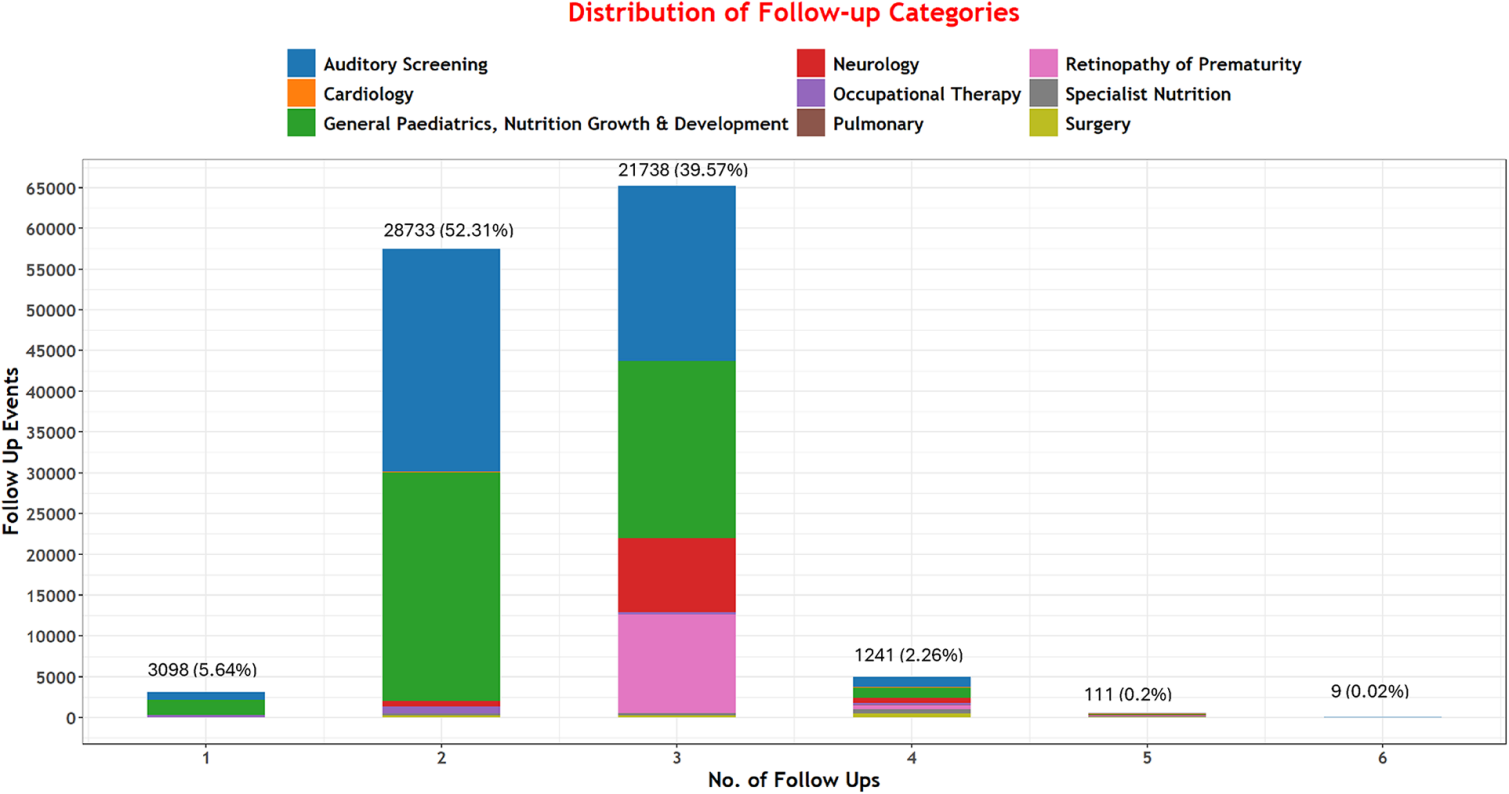
Distribution of follow-up services.

Among 136,249 neonates discharged, 54,930 (40.3%) met the criteria for at least one specialist follow-up. Among these, we project that 131,351 specific follow-up episodes would be needed across our nine specified categories. Of those needing follow-up, very few met the criteria for only one category; many met the criteria for two (52.3%, 28,733) and three (39.6%, 21,738) follow-up episodes Figure 2.

Other than a general pediatric, nutrition, growth, and development follow-up episode, most babies required follow-up for auditory screening, retinopathy of prematurity/ophthalmology, and neurology concerns (50,992 (38.8%), 12,616 (9.6%), 10,452 (8%)) respectively Table 3.

### Follow-up categories and contributing morbidities

The factors or conditions contributing to specific categories of follow-up needs are presented in Table 4. Among the 8% of the discharged newborn population with neurology needs, two factors contributed to 88% of cases: severe birth asphyxia (SBA) and/or HIE, and seizures. Additionally, the need for review to assess for ROP, at 9.6% of survivors, was mostly (99%) associated with the risk factor of having a BW of 1,500 g or less. Approximately 86% of the 50,992 cases that needed auditory screening were individuals who had experienced severe infections treated with gentamicin for four or more days, jaundice managed with phototherapy, and those born weighing 1,500 g or less Table 4.

**Table 4:** Neonatal morbidities/phenotypes for the respective follow up categories.

(A)					
Neurology	*N* (%)	Specialist nutrition	*N* (%)	Auditory screening	*N* (%)
SBA/HIE Convulsions/seizures Neonatal meningitis Neonatal tetanus Paralysis Kernicterus	5,568 (46.8%) 4,932 (41.4%) 1,067 (9%) 288 (2.4%) 45 (0.4%) 9 (0.1%)	Facial/Lip Cleft Necrotizing Enterocolitis-NEC Gastroschisis Hirschsprung	412 (36.3%) 372 (32.8%) 311 (27.4%) 39 (3.4%)	Severe infections Phototherapy Birth weight ≤1500g SBA/HIE Convulsions/seizures RH/ABO Isoimmunization Neonatal Meningitis Facial/Lip Cleft Neonatal Tetanus Kernicterus	35,722 (37.6%) 33,115 (34.9%) 12,575 (13.2%) 5,568 (5.9%) 4,932 (5.2%) 1,295 (1.4%) 1,067 (1.1%) 412 (0.4%) 288 (0.3%) 9 (0%)
(B)					
General pediatrics, nutrition, growth & development	*N* (%)	Retinopathy of prematurity & ophthalmology	*N* (%)	Pulmonary	*N* (%)
Neonatal Jaundice Birth weight ≤1500g SBA/HIE Convulsions/seizures Birth/Tissue Injury Hypoglycaemia Congenital Deformities Facial/Lip Cleft Kidney Injury Necrotizing Enterocolitis-NEC Down Syndrome Spina Bifida Gastroschisis Exchange transfusion Patent Ductus Arteriosus Hernia Pulmonary hypertension Bronchopulmonary Hirschsprung hydronephrosis Mechanical ventilation Kernicterus	33,636 (53.8%) 12,575 (20.1%) 5,568 (8.9%) 4,932 (7.9%) 1,232 (2%) 931 (1.5%) 649 (1%) 412 (0.7%) 385 (0.6%) 372 (0.6%) 368 (0.6%) 317 (0.5%) 311 (0.5%) 258 (0.4%) 248 (0.4%) 179 (0.3%) 65 (0.1%) 63 (0.1%) 39 (0.1%) 14 (0%) 11 (0%) 9 (0%)	Birth weight ≤1,500 g/GA <32 weeks Bronchopulmonary dysplasia Mechanical ventilation Kernicterus	12,575 (99.3%) 63 (0.5%) 11 (0.1%) 9 (0.1%)	Bronchopulmonary dysplasia Mechanical ventilation	63 (85.1%) 11 (14.9%)
(C)					
Occupational therapy	*N* (%)	Cardiology	*N* (%)	Surgery	*N* (%)
Birth injury CTEV Paralysis	1,232 (70%) 483 (27.4%) 45 (2.6%)	Patent ductus arteriosus Pulmonary hypertension	248 (79.2%) 65 (20.8%)	Facial/Lip Cleft Spina Bifida Gastroschisis	412 (39.6%) 317 (30.5%) 311 (29.9%)

GA, gestation age.

Among the newborn population needing general pediatric, nutrition, growth, and development follow-up, jaundice (*moderate and severe*, *treated with phototherapy*), and very low BW (≤1,500 g) were by far the most common indications (74%) with important sub-groups resulting from SBA/HIE (9%), seizures (8%), birth injury (2%) and hypoglycemia (1.5%).

Survivors requiring early occupational therapy follow-up were mostly related to birth/tissue injury (70%). Currently routine records in these settings with limited diagnostic capacity suggest only 0.1% of survivors and 0.2% of survivors would need specific pulmonary or cardiology follow-up respectively. Diagnoses of BPD (85.1%) and Patent Ductus Arteriosus (PDA, 79.2%) are the major indications.

## Discussion

The reduction of neonatal mortality has been a significant area of focus, leading to remarkable advancements in the survival rates of small and sick newborns. Survivors may carry a burden of chronic disorders and disabilities into childhood and adulthood with considerable societal costs. An absence of diagnostic equipment and skilled personnel in LMIC such as Kenya, means we remain very uncertain of the prevalence of specific morbidities such as visual and hearing impairment, cognitive deficits, learning disabilities, and behavioral challenges. Our study was not able to strengthen diagnostic capability to identify specific impairments but endeavored, within a large cohort, to shed light on the likely early follow-up needs of nearly 131,000 NBU survivors. To do this we adopted a pragmatic approach, utilizing available clinical characteristics to identify phenotypes and assign newborns to early post-discharge follow-up categories. It is important to note that formal follow-up programs in Kenya are not readily available, except for some at the tertiary hospital. Currently, in hospitals providing intermediate-level neonatal care, only those with the most evident indications for follow-up (e.g., explicit congenital, neurological, or other abnormalities) are likely to be prioritized.

Our data suggest individual babies often need multiple types of follow-up services. General pediatric and nutrition, growth and development, auditory screening, and ophthalmology/ROP are the three services likely to be most in demand. These essential services extend beyond hearing and visual screening follow-ups mainly outlined in the “WHO recommendations on maternal and newborn care for a positive postnatal experience”, aligning with the indicated needs of these infants (21). These findings highlight the importance of providing a coordinated set of services. For instance, auditory and ROP screenings could be bundled and delivered by a multi-professional team and offered at the same return hospital visit as a priority. Such support would be most family-friendly, reducing time requirements, out-of-pocket costs, and ultimately help reduce the number of different follow-ups needed and decrease the risk of patients lost to follow-up. Such a coordinated set of services could mean that 9 out of 10 of the approximately 40% of NBU survivors in our cohort that need two or three of the commonest forms of follow-up might achieve this in a single initial visit to the hospital (see Table 3 and Figure 2).

Roughly 1–3 in 1,000 full-term babies have lasting hearing loss, escalating to 2–4 in 100 for high-risk infants such as those requiring intensive care (22–24). Prolonged gentamicin use can heighten the risk of hearing-related complications due to ototoxicity (25) while bilirubin's toxic effects can lead to early sensorineural hearing loss (26–28). Early detection facilitates language development, communication skills, and caregiver relationships (23, 26, 29). The repercussions of undetected and untreated hearing loss go beyond negative effects on speech and language outcomes, particularly in LMICs (30). Newborn hearing screens for all newborns within one month of life are currently the standard of care in many HIC (31) and are the recommendation by the WHO (30). However, universal auditory screening for all newborns presents challenges in resource-limited settings due to the inadequacy of requisite resources (25). Our approach suggests that almost 40% of NBU survivors might require follow-up for auditory screening as *a priori*ty, assuming no routine, pre-discharge screening is feasible. Very low birth weight, infections treated with gentamicin for greater than four days (with no therapeutic drug monitoring) and jaundice/hyperbilirubinemia were the most common indications for such screening in our cohort.

Approximately 10% of those discharged from the study hospitals’ NBUs would require screening for retinopathy of prematurity (ROP), which remains a leading cause of childhood visual impairment and blindness (32, 33). In reality, we expect this percentage of infants requiring ROP screenings to increase based on the higher screening BW and GA criteria used in some LMICs compared to the criteria we used for the analysis, and that of even lower threshold used in HICs (34, 35) This is due to the fact that infants with higher BW and GA in LMICs in comparison to HICs develop ROP secondary to unregulated oxygen use (36). Based on clinical diagnoses and documentation of interventions received for ROP, screening needs appear to be highly variable across sites in our study, from as low as one (1) to as much as 121 per year in another, probably reflecting lack of universal screening infrastructure and highlights the need of additional ROP services throughout the county hospitals. One option to increase screening may be to bundle and regionalize screening services for auditory and ophthalmology screenings. Although this would concentrate skills and resources in selected centers, it may have impact on equity of access and delay screening and treatment in urgent cases as it would be offered after discharge. Alternatively, advances in technology might allow for task-shifting combined with telemedicine to offer broader access while in the hospital setting.

Monitoring neurological abnormalities and developmental milestones after birth is crucial, particularly in LMICs where neurological and developmental delay cases are common (37). In these settings, developmental disability prevalence is reported to vary widely, ranging from 0.4% to 45.2% (37). For instance, in Uganda, Namazzi et al. (2019) found a 12.7% prevalence of at least one delay associated with perinatal asphyxia and post-neonatal complications (37). Bitta et al. (2018), in a systematic review encompassing population-based studies assessing the prevalence or incidence of neurological and neurodevelopmental disorders, reported a prevalence rate of 11.3 per 1,000 population for neurological disorders among children under 19 years in LMICs (38). Our study suggests that 8.0%, (80 per 1,000) NBU survivors should probably be offered initial specific neurodevelopmental follow-up. This rate is relatively high, considering that it pertains to the entire inpatient neonatal care unit population based although this is based on identified risk factors rather than confirmed diagnoses. Severe perinatal asphyxia necessitates close nutritional and developmental evaluations encompassing vision, hearing, language, and cognitive skills (39–41). A comprehensive multidisciplinary team comprising specialists in pediatric neurology and developmental pediatrics is essential for evaluating developmental milestones, and neurological progress, and diagnosing conditions like neuromotor impairments and cerebral palsy (41). Improving these services would facilitate early detection and interventions, addressing what may be a growing burden of disability in these communities.

Early-life nutrition significantly shapes infant growth and development, with potential long-term impacts on childhood and adulthood as vital organs and systems mature (42). Especially for preterm infants, the primary aim is achieving optimal growth and development from the onset, recognizing that malnutrition has onset in early infancy in many cases (43). Most VLBW infants leave Neonatal Care Units undernourished and inadequately grown, particularly affecting breastfed preterm VLBW infants (44, 45). Prematurity complications heighten the risks of postnatal growth failure (39, 40). Such infants require more specific follow-up than is offered through routine growth monitoring provided in combined Mother and Child Health Wellness and Immunization clinics. This follow-up would ideally be provided by teams comprising pediatricians/neonatologists and nutritionists as part of a high-risk follow-up clinic for small and sick neonates. This type of high-risk clinic is currently not mandated in Kenya and would be different from the currently practiced well-child clinics typically run by nurses with or without a nutritionist.

Specialized nutrition requirements are crucial for infants with unique medical conditions. For infants with facial or lip clefts, occurring in 0.28–3.74 in 1,000 live births (46), tailored feeding techniques involving specialized bottles or nipples aid in safe and efficient feeding, reducing the risk of aspiration and promoting adequate nutritional intake (46, 47). Infants recovering from necrotizing enterocolitis (NEC), estimated to have a global prevalence of around 7% among those categorized as VLBW (≤1,500 g) (48), may benefit from gradual feeding reintroduction or specialized formulas to support digestive healing while supporting transition to enteral feeding for stimulated growth (48). Survivors of gastroschisis, who are at increased risk for impaired growth, often require carefully managed feeding plans, transitioning from parenteral to enteral feeds for optimal digestive recovery post-operation (49). Additionally, infants following Hirschsprung disease may need closely monitored feeding schedules to address their bowel function challenges effectively (50). These specialized nutritional approaches would play a pivotal role in supporting the health and well-being of infants with these diverse medical conditions. Furthermore, growing survivors of these conditions in LMICs again highlights the need for a comprehensive follow-up program with focus on nutrition and neurodevelopmental outcomes.

Some common follow-up needs among discharged newborns may be feasible to address in hospitals with moderate volume intermediate care Neonatal Care Units (NBUs). However, specific needs might require concentration in fewer locations or alternative approaches like referral team visits. Careful planning is necessary to ensure access across regions, avoiding exclusive provision in capital city tertiary units. These follow-up requirements are inherently multidisciplinary, demanding a team of experts comprising paediatricians/neonatologists, child psychologists, pediatric neurologists, ophthalmologists, audiologists, occupational therapists, social workers, and dieticians under one roof (3). Long-term support, including counseling for families and effective communication among all team members, is paramount (51). It is daunting to plan for an ideal follow-up clinic encompassing the many required specialists without the hurdle of a healthcare systems change, as implementing such comprehensive follow-up programs will significantly increase the workload and utilization of already scarce resources. This is already challenging with typically just 1–2 pediatricians per hospital handling NBU, pediatric wards, routine outpatient departments, and administrative duties (19). However, as demonstrated by the distribution of follow-up needs we outline, we propose bundling follow-up services as a starting step. General pediatrics/nutritional assessment combined with auditory screening was the most needed follow-up. Additionally, targeting specific higher risk groups such as the infants born <1,500 g/32 weeks for coordinated care could be ideal, especially in the setting of creating high-risk follow-up clinics. Incorporating auditory and ROP screenings to these visits would allow for detection and prevention for deafness and blindness in the highest risk infants. Ultimately, it will necessitate addressing the broader issue of workforce planning and service design, which is intricately linked to the decentralization or centralization of services. This aspect necessitates a policy discussion as it carries major funding implications for equity and access.

In Kenya, there remains a significant gap in documenting and understanding the capacity of local staff and infrastructure to manage specialized follow-ups for vulnerable infants. Anecdotal evidence suggests considerable variation across different levels of care. For instance, a study by Jayawardena et al. (2018) highlighted the scarcity of resources, indicating that Kenya had only 0.12 audiologists per 1,000,000 people, in stark contrast to the 41 audiologists per 1,000,000 people in practice in the UK (52). This underscores the pressing need for further investigation and improvement in the capacity of local staff and infrastructure to adequately address the needs of vulnerable infants requiring specialized follow-up care.

## Limitations

Limitations inherent in our study stem from its retrospective design and heavy reliance on medical records, which are often prone to incomplete or suboptimal documentation in routine care settings. This reliance on clinical data, together with limited diagnostic capacity, might have led to few reported cases requiring pulmonary or cardiology follow-ups among others. Additionally, discharge summaries and documentation of discharge diagnoses are often done by junior clinicians in these settings, which might introduce discrepancies, variability, and reliability concerns. We also included cases from both one tertiary hospital and 22 intermediate newborn care centers, potentially causing variations in the types of cases encountered across these different levels of care. The process of categorizing survivors’ phenotypes and assigning follow-ups, while pragmatic, was conducted by only a small group of experts, others would potentially have made different conclusions. Given these challenges, a cautious interpretation of the study's outcomes and conclusions is needed although we believe this report is an important first step in highlighting what may be minimum follow-up needs amongst NBU survivors in a set of LMIC hospitals.

Additionally, the lack of empirical data on the availability of specialists and post-discharge care guidelines limited our ability to fully contextualize the identified needs within the healthcare system's capacity. While our study primarily aimed to identify these needs, we acknowledge that estimating patient-to-specialist ratios and assessing the healthcare system's capacity to meet these needs could offer valuable insights beyond the scope of our current investigation. Future studies should therefore focus on gathering empirical data on specialist availability and developing comprehensive post-discharge care guidelines to address these gaps effectively. Despite these limitations, our study serves as a foundational step towards further understanding and addressing the comprehensive follow-up needs of vulnerable newborns. We advocate for future works to prioritize consensus-building among practitioners to establish frameworks that balance desirability and feasibility in defining post-discharge review guidelines, timelines, policy design, and health service organization, taking into account considerations for both centralization and decentralization of services across various levels of care.

## Conclusion

This study illuminates the significant yet largely unmet need for early follow-up care among small and sick newborns who survive NBU admission in Kenya. It highlights a considerable gap between the necessary follow-up services required for these vulnerable newborns and the current services available, as far as our knowledge extends. This disparity is compounded by the intricate challenges of organizing and coordinating healthcare services, including the absence of specific coordination roles and functions, the lack of local guidelines on newborn/infant discharge and post-discharge processes, and a shortage of trained professionals to provide these services.

Of notable concern is that over 40% of these survivors are anticipated to require specific follow-up care, often encompassing general pediatric care intertwined with developmental and auditory screening. Implementing a holistic approach to service delivery at both the hospital and community levels could significantly benefit babies and their families. Such comprehensive follow-up services are integral to realizing infants’ full potential as part of the “survive and thrive” agenda.

While centralizing certain services may initially enhance efficiency, it raises concerns about equity, especially considering the limited access to provider teams with the requisite skills in many facilities and communities. Thus, the efficient development and delivery of follow-up care strategies tailored to the needs of resource-limited settings like Kenya become paramount for service development and equitable healthcare provision.

## Data Availability

The datasets generated and/or analysed during the current study are not publicly available due to the primary data being owned by the hospitals and their counties with the Ministry of Health. The research staff do not have permission to share the data without further written approval from both the KEMRI-Wellcome Trust Data Governance Committee and the Facility, County or Ministry of Health as appropriate to the data request. Data used for this article are available in Harvard Dataverse at: https://doi.org/10.7910/DVN/ZX7VQK. Access applications can be made through the Data Governance Committee with details available on www.kemriwellcome.org, or an email to dgc@kemri-wellcome.org. Requests for access to primary data from quantitative research by people other than the investigators will be submitted to the KEMRIWellcome Trust Research Programme data governance committee as a first step through emailing dgc@kemri-wellcome.org who will advise on the need for additional ethical review by the KEMRI Research Ethics Committee.

## Collaborators

Clinical Information Network authors: the CIN author group who contributed to the network's development, data collection, data management, implementation of audit and feedback and who reviewed and approved this publication includes: NEST360 Kenya & Aga Khan University: Dolphine Mochache. Vihiga County Hospital: Juma Vitalis, Arnest Namayi. Kakamega County Referral Hospital: Nyumbile Bonface, Dolphine Mochache, Samuel Soita. Mbagathi County Hospital: Christine Manyasi, Jane Mbungu, Joan Baswetty. Mama Lucy Kibaki Hospital: Caren Emadau, Josephine Aritho, Beatrice Njambi, Esther Mwangi. Machakos County Hospital: Charles Nzioki. Nyeri County Referral Hospital: Wagura Mwangi, Lucy Kinyua, Alfred Wanjau. Kisumu East County Hospital: Magdalene Kuria, Alice Oguda, Otieno George Obop. Embu County Referral Hospital: Esther Njiru, Pauline Njeru. Kerugoya County Hospital: Penina Mwangi, Nancy Mburu, Mary Nguri. Kitale County Hospital: Rachel Inginia, Celestine Muteshi, Jeska Kuya. Busia County Hospital: Emma Namulala, Salome Okisa Muyale, Susan Wanjala. Kiambu County Hospital: Grace Ochieng, Lydia Thuranira, Faith Mueni, Margaret Waweru. Bungoma County Referral Hospital: Felistus Makokha, Rosemary Mututa. Jaramogi Oginga Odinga Teaching and Referral Hospital: Josephine Ojigo, Joyce Oketch. Pumwani Maternity Hospital: Beth Maina, Orina Nyakina, Faith Njeru, Judith Onsongo. Thika County Referral Hospital: Bernadette Lusweti, Margaret Wanjiku Mwaura, Peter Muigai. Vihiga County Referral Hospital: Amilia Ngoda. Machakos County Referral Hospital: Zainab Kioni, Esther Muthiani. Embu Teaching & Referral Hospital: Loise N. Mwangi. Bungoma County Hospital: Seline Kulubi. JOOTRH: John Ollongo. Mbagathi District Hospital: Joseph Ng’ang’a. Nakuru Teaching and Referral Hospital: Benjamin Tanui.
